# From novice to expert: a qualitative study of implementation facilitation skills

**DOI:** 10.1186/s43058-020-00006-8

**Published:** 2020-02-25

**Authors:** Mona J. Ritchie, Louise E. Parker, JoAnn E. Kirchner

**Affiliations:** 1 VA Quality Enhancement Research Initiative (QUERI) Program for Team-Based Behavioral Health, Department of Veterans Affairs, 2200 Ft Roots Dr, Building 58, North Little Rock, AR 72114 USA; 2 Department of Psychiatry, University of Arkansas for Medical Sciences, 4301 W Markham St, #755, Little Rock, AR 72205 USA; 3 Department of Management and Marketing, College of Management, University of Massachusetts, 100 Morrissey Blvd, Boston, MA 02125 USA

**Keywords:** Facilitation, Implementation, Skills

## Abstract

**Background:**

It is widely reported that facilitation can aid implementation of evidence-based practices. Although scholars agree that facilitators need a diverse range of skills, only a few retrospective studies have identified some of these. During the test of a facilitation strategy within the context of a VA initiative to implement evidence-based care delivery models, we documented the skills an expert external facilitator transferred to two initially novice internal regional facilitators. Ours is the first study to explore facilitation skills as they are being applied and transferred.

**Methods:**

Facilitators applied the strategy at eight primary care clinics that lacked implementation capacity in two VA networks. We conducted monthly debriefing interviews over a 30-month period and documented these in detailed notes. External facilitator interviews focused specifically on training and mentoring internal facilitators and the skills that she transferred. We also conducted, recorded, and transcribed two qualitative interviews with each facilitator and queried them about training content and process. We conducted a content analysis of the data, using deductive and inductive methods, to identify skills the external facilitator helped internal facilitators learn. We also explored the complexity of facilitation skills and grouped them into overarching skillsets.

**Results:**

The external facilitator helped internal facilitators learn 22 complex skills; with few exceptions, these skills were not unique but overlapped with one another. We clustered 21 of these into 5 groups of overarching skillsets: (1) building relationships and creating a supportive environment, (2) changing the system of care and the structure and processes that support it, (3) transferring knowledge and skills and creating infrastructure support for ongoing learning, (4) planning and leading change efforts, and (5) assessing people, processes, and outcomes and creating infrastructure for program monitoring.

**Conclusions:**

This study documented a broad range of implementation facilitation skills that are complex and overlapping. Findings suggest that studies and initiatives planning or applying facilitation as an implementation strategy should ensure that facilitators have or have the opportunity to learn the skills they need. Because facilitation skills are complex, the use of didactic methods alone may not be sufficient for transferring skills; future work should explore other methods and techniques.

Contributions to the literature

- Implementation facilitation is increasingly used to help healthcare settings implement evidence-based innovations.
- Although there is consensus that facilitators need a wide range of skills, few empirical studies have identified or described facilitation skills. Our study fills this gap in the literature.
- We identified and described 22 implementation facilitation skills that an expert facilitator applied and transferred to initially novice internal healthcare system change agents. All of the skills were complex, and most overlapped with other skills.
- Our findings can inform the transfer of facilitation skills in research studies and clinical initiatives to ensure fidelity to planned implementation facilitation strategies.

## Background

Implementing and sustaining evidence-based practices, indeed any innovation, is frequently challenging [1–3]. To successfully implement innovations, organizations need to involve stakeholders from multiple organizational levels [3–5], apply a variety of implementation strategies tailored to local context, leverage existing enablers, and address barriers to change [6–9]. Unfortunately, not all organizations have the capacity for conducting change on their own; many lack infrastructure support, necessary resources, or knowledge and understanding of change processes [2, 3, 10, 11]. Facilitation has been widely utilized in research studies and clinical initiatives to help such healthcare settings successfully implement evidence-based practices and programs, prevention services, and complex models of care delivery [12–17]. In fact, the integrated Promoting Action on Research Implementation in Health Services (iPARIHS) framework posits that facilitation is the *active* ingredient in implementation efforts [18]; facilitators address factors related to the innovation, the innovation recipients, and the context that can hinder or foster implementation. Transferring implementation facilitation skills to internal change agents could help healthcare systems improve their ability to implement evidence-based practices and programs [15, 16]. However, understanding what those skills are, the focus of this article, is a prerequisite to understanding how to transfer them.

Although there are multiple definitions of facilitation [12, 13, 19], the most frequently utilized ones describe facilitation as a multimethod process for easing the adoption, implementation, and sustainability of new practices [20]. Facilitation strategies are complex. Skilled facilitators, who may be external and/or internal to the setting, assess targets of the implementation effort, characteristics of the innovation, and the status and needs of the organizational context as well as implementation progress and barriers to implementation [20]. They apply a wide variety of techniques and processes [13, 21, 22] and tailor them to the needs and resources of the healthcare setting [22] to counter the multiple factors that hinder implementation and foster those factors that support it. Because implementation processes are dynamic and change over time, facilitators respond to those changes as well as changes in the local and organizational environments [21, 23]. Additionally, change often has to occur at all levels to improve quality of care across the healthcare system [4, 24]; many facilitators assess and intervene across most, if not all organizational levels. Finally, facilitators moderate the strength of their interventions as not all settings need intensive facilitation [25], and at times, even limited facilitation can significantly improve delivery of care [26, 27].

Given the complexity of implementation facilitation, it is not surprising scholars suggest that facilitators need a diverse range of knowledge and skills that they can apply depending on the purpose and context of their efforts [13, 28–30]. Lack of these skills may compromise fidelity to the planned facilitation intervention and negatively influence outcomes [31–34]. Thus, studies or clinical initiatives applying facilitation as an implementation strategy need to ensure that facilitators have the necessary skills or provide training for developing those skills [35, 36]. Although lists of the types of skills facilitators need exist in conceptual literature, project descriptions, and reviews [20], there are only a few empirical studies that documented implementation facilitation skills. Further, in these studies, skills were documented retrospectively; researchers asked facilitators what skills they had needed in previous implementation efforts [28, 37, 38]. Ours is the first study to document implementation facilitation skills as they are being applied and transferred.

We conducted the study within the context of a Department of Veterans Affairs (VA) initiative, including a mandate and national-level resources for primary care clinics to implement evidence-based primary care mental health integration (PCMHI) care models, and a large VA-funded project that applied and evaluated the role of facilitation for supporting PCMHI implementation. This project applied the facilitation strategy in eight VA primary clinics, conducted an independent evaluation of the strategy, and compared outcomes to eight clinics that did not receive facilitation [15, 16]. National resources available to all clinics included consultation, technical assistance, education and training, and informational tools [39]. The facilitation strategy, which was informed by the original PARIHS framework [29, 40], supported PCMHI implementation in the primary care clinics and transferred facilitation skills to internal regional facilitators who became experts in implementation facilitation, thereby building capacity in their regions for future implementation efforts. This study, a component of the larger project and the first author’s doctoral dissertation [41], explores the range of skills the expert transferred and how facilitators operationalized them.

## Methods

### Study setting

We conducted the larger project in eight primary care clinics, four in each of two VA regional networks. We selected the VA networks based on (1) their ability to identify an internal regional facilitator who would support PCMHI implementation at 50% effort and (2) their willingness to participate in the study. Mental health leaders in each of the two networks then identified one VA medical center and three community-based outpatient clinics of varying sizes where the primary care clinics planned to implement PCMHI in the first year of the study but would have difficulty without implementation assistance.

### The implementation facilitation intervention

An external facilitator, JEK, with expertise in PCMHI care models, implementation science, and facilitation and one internal regional facilitator in each of the two VA networks applied the strategy. Both internal facilitators were network-level employees. One was a clinical psychologist and the other a social worker; neither internal facilitator initially had implementation science or facilitation expertise. Network mental health leaders identified staff for the internal facilitator roles, and the external facilitator worked with the internal facilitators from May and July 2009 to the fall of 2011. Over the course of the study, the facilitators applied a wide variety of strategies to help primary care clinics implement PCMHI. Thorough descriptions of their efforts have been well-documented elsewhere [15, 16]. A key component of the external facilitator’s role was to help the internal facilitators learn implementation facilitation knowledge and skills so that over time, internal facilitators could become experts in implementation processes and how to facilitate them.

### Data collection

For the larger project, two highly experienced female qualitative researchers conducted debriefing interviews with the external facilitator and initially novice internal facilitators. Both interviewers had personal relationships with the external facilitator (JEK). The primary interviewer (LEP) was an organizational scientist, and the second interviewer (MJR) earned a PhD in Public Policy during the study. We conducted joint debriefings with the external facilitator and the relevant internal facilitator immediately after the initial visit to each study site. We then conducted monthly individual, approximately hour long, debriefings with all three facilitators from August 2009 to November of 2011. During external facilitator debriefings, we focused primarily on activities intended to help internal facilitators learn how to facilitate implementation. During internal facilitator debriefings, we tracked the ongoing facilitation process and implementation progress at each site, and we collected information relevant to the external facilitator’s training of internal facilitators. In total, we conducted 85 debriefing interviews by telephone. Due to the large amount of other qualitative data collected for the project, we did not have the resources to transcribe recordings of these debriefings. We thus took extensive notes and documented facilitators’ responses as close to verbatim as possible. One interviewer drafted the notes, and both reviewed and came to consensus on their content or followed up with the facilitator to obtain clarification.

We also conducted 1- to 2-hour semi-structured qualitative interviews by telephone with each of the facilitators using an interview guide to assess the implementation facilitation and skills transfer processes approximately 16 months after the initial visit to study sites and again at the end of the study. The same researchers conducted these interviews. We audio-recorded the interviews and produced verbatim transcripts from the recordings. In total, we conducted six interviews with facilitators.

Finally, we took notes to document facilitators’ interactions during meetings to prepare for initial in-person site visits. Qualitative data collected for the larger project, including debriefing notes, transcripts for semi-structured qualitative interviews, and pre-site visit call notes, served as source data for the current study. The VA Central Institutional Review Board (#09-05) approved the conduct of the larger project, including the documentation of facilitator’s quality improvement activities in debriefing and call notes. We conducted a formal verbal consent process with the two internal facilitators prior to conducting the qualitative interviews described above.

### Data analysis

For this study, using ATLAS.ti (2016), MJR and LEP conducted a mix of directed and conventional content analysis [42, 43] of the source data described above. Directed content analysis typically uses theory or prior research to identify coding categories with a goal of validating or extending what is known [43, 44]. Although there are few empirical studies documenting facilitation skills, some researchers have described skills that they believe facilitators had or needed; this type of information also exists in publicly available facilitation training materials. We reviewed this literature and materials and found wide variability in the naming and description of skills. For example, where some authors suggested facilitators need communication skills [38, 45], others suggested they need specific communication skills, e.g., good listening skills [21], presentation skills [46], or the ability to put arguments across [47].

To develop the code list, we first identified types of skills listed in literature and other materials (see Additional file 1). We then reviewed all of the source data for those skills, refining the code names and definitions based on source material. MJR then applied the codes; added four new ones, in consultation with LEP, as they emerged from the data via a conventional content analysis; and refined codes throughout the analysis process. We inductively identified themes in the coded material related to the operationalization of each skill and then developed a summary of the behaviors related to that skill.

Using ATLAS.ti’s network function which displays linkages between constructs, in this case skills, allowed us to examine relationships between skills [48]. For each skill, MJR applied an intensive iterative process of data analysis. Specifically, for each skillset, she:

- Reviewed the text describing instances in which the external facilitator was helping internal facilitators learn a particular skillset to develop a thorough understanding of how the external facilitator operationalized the selected skillset (e.g., interpersonal skills).
- Reviewed the text for each of the other 21 skills to evaluate whether any of these could be a subset of the selected skillset (e.g., could interpersonal skills be operationalized without also using communication skills?).
- Documented the overlap by connecting skillsets in ATLAS.ti when another skillset was needed to operationalize the selected skillset.

Finally, in consultation with LEP, she inductively clustered or grouped 21 of the skills into categories [48] based on the types of facilitation processes the external facilitator was helping internal facilitators to learn, resulting in 5 overarching, higher-level skillsets. Communication skills did not fit into any of these skill clusters as it seemed to be a unique set of skills; although communication skills were part of other skills, other skills were not part of communication skills. She then revised the display of linkages between skills to account for the skill clusters. The third author, who was the external facilitator, and one of the internal facilitators reviewed and confirmed all findings.

## Results

We identified 22 implementation facilitation skillsets that the external facilitator transferred to internal facilitators. Communication skills seemed to be a crosscutting skillset that was a part of and needed to apply many of the other skills. We clustered the other 21 skills into 5 groups of overarching higher-level skillsets. (1) *Building relationships and creating a supportive environment* included the skills needed to interact with stakeholders across organizational boundaries and levels in order to motivate and foster their support of and participation in implementation and sustainment. In transferring implementation facilitation skills to internal facilitators, the external facilitator focused most frequently on communication skills and the skills in this set. (2) *Changing the system of care and the structure and processes that support it* included skills for helping stakeholders design and adapt the program to meet local needs and resources, integrate it into the organization, identify and address challenges, and use data to improve the program and its implementation. (3) *Transferring knowledge and skills and creating infrastructure support for ongoing learning* included the abilities to provide information and help stakeholders learn the skills needed for providing PCMHI services as well as continuing the learning process in the future. (4) *Planning and leading change efforts*, in addition to administrative and project management skills, included the abilities to think strategically and lead and manage team processes. (5) *Assessing people, processes, and outcomes and creating infrastructure for program monitoring* included the ability to help stakeholders identify and/or develop measures and processes for assessing and monitoring program implementation and outcomes. We also described each of the skills, including some information about how they were operationalized. For example, our description of interpersonal skills includes listening to stakeholders and ensuring they have opportunities to express themselves, assessing and addressing their needs and concerns, and knowing when and how to be assertive and still be supportive. See Table 1 for the list of skills within clustered skillsets and descriptions of each.

**Table 1:** Implementation facilitation skills, descriptions, and related skills

Facilitation skills	Descriptions of skills	Skills that are included
Communication skills	Interacting with individuals and groups, orally or in writing, to share information, e.g., through formal presentations, less formal conversations, emails, messages, and reports; listening to stakeholders; and asking questions to understand their needs and concerns	None
Skill group 1: Building relationships and creating a supportive environment		
Interpersonal skills	Interacting with stakeholders in positive ways, e.g., listening to stakeholders and ensuring they have opportunities to express their opinions, deferring to them when appropriate, working around their schedules, assessing and addressing their needs and concerns, and knowing when and how to be assertive and still be supportive	Assessment, communication
Stakeholder engagement	Involving stakeholders (individuals/teams that can affect or will be affected by the innovation) and fostering participation in planning and implementation processes, as well as tailoring interactions to their needs	Assessment, communication, education and marketing, interpersonal, motivating and building confidence, political
Motivating/building confidence	Praising stakeholders for participation and implementation progress and encouraging them to assess their own efforts, share their successes, solve problems, and create their own strategies	Communication, interpersonal
Political skills	Assessing, understanding, navigating, and leveraging the political dynamics of the setting	Assessment, communication, interpersonal, problem identification/solving
Interacting and working with leaders	Combining and applying all of the skills in this group to obtain the support and involvement of leaders, includes being comfortable with leadership at all levels, adopting a power stance when appropriate, and being respectful of leaders’ time and supportive of their decisions	Communication, stakeholder engagement, interpersonal, motivating and building confidence, political, pulling back and disengaging
Skill group 2: Changing the system of care and structures and processes that support it		
Helping to design/adapt a program to meet local needs	Helping stakeholders plan a PCMHI program that fits with local needs and available resources and further adapt the program based on implementation progress and outcomes data and emerging barriers and enablers	Assessment, communication, interpersonal, leading and managing team processes, presenting and using data, problem identification/solving
Problem identification and solving skills	Identifying and addressing problems and helping stakeholders identify and address problems, e.g., lack of space, implementation resources, leadership support, and stakeholder participation	Assessment, presenting and using data, stakeholder engagement
Presenting/using data to improve the program	Reviewing, interpreting, and presenting qualitative and quantitative information and using this information, e.g., to support and encourage stakeholder efforts, plan interventions to improve implementation, and support problem identification	Communication, interpersonal, monitoring implementation, stakeholder engagement, thinking strategically and planning
Helping integrate the program into other programs/services	Identifying and collaborating with leaders/staff of programs whose patients might need PCMHI services, who may provide additional services for PCMHI patients, or who may benefit from knowledge of the PCMHI program, to support sustainability after active implementation	Assessment, communication, interpersonal, leading and managing team processes, thinking strategically and planning
Skill group 3: Transferring knowledge and skills and creating infrastructure support for ongoing learning		
Education/marketing skills	Persuasively presenting and discussing PCMHI models, how they work, and the evidence for them; their value, benefits, and outcomes; and how to implement them, including how to address implementation challenges, as well as tailoring content and process to stakeholder needs and concerns	Assessment, communication, interpersonal
Training, mentoring, and coaching skills	Using training, mentoring, and coaching techniques to transfer skills to clinicians and leaders for providing PCMHI services, e.g., for delivering evidence-based PCMHI care models, rather than traditional mental healthcare, and increasing the number of PCMHI patients	Assessment, communication, education and marketing, learning and fostering learning, monitoring implementation
Learning and fostering learning skills	Applying learning strategies (e.g., learning from experts, others similar to yourself, and from past experiences) to fill in gaps in knowledge and build on existing knowledge and skills, and fostering stakeholder use of these strategies	None
Building learning collaboratives	Building a learning collaborative for PCMHI clinicians to support implementation by encouraging participation, facilitating meetings/calls, and encouraging members to share their own experiences and problems, work on solutions, and develop best practices	Communication, education and marketing, interpersonal, learning and fostering learning, pulling back and disengaging
Skill group 4: Planning and leading change efforts		
Administrative and project management skills	Performing technical tasks, e.g., working with sites to plan and schedule site visits and conference calls and disseminating materials and site visit reports, and pushing implementation forward when stakeholders/sites were not responding, or implementation processes were stalled	Interpersonal, monitoring implementation, political, and problem identification/solving
Meeting facilities and individuals where they are	Accepting and working with site and stakeholder limitations, building on their strengths, and helping them be as successful as possible	Interpersonal
Leading/managing team processes	Facilitating communication and managing conflict/disruptive behavior; guiding team processes, e.g., by sharing ideas, affirming stakeholder input, fostering team self-management; and leading task-oriented processes, e.g., goal setting, program design and adaptation, decision-making, and problem identification and solving	Assessment, communication, interpersonal, problem identification/solving, stakeholder engagement
Thinking strategically and planning	Thinking through what was currently happening at sites, what needed to happen for successful implementation, and how facilitators could help; planning/preparing for implementation events; and diagnosing/evaluating sites and implementation processes	Administrative and project management, assessment, monitoring implementation, problem identification/solving
Pulling back and disengaging	Gaging when stakeholders are ready to assume responsibility for implementation efforts and refraining from acting as the expert, deferring decision-making to leaders, helping stakeholders explore options and come to consensus, and saying good-bye	Assessment, interpersonal
Skill group 5: Assessing people, processes, and outcomes and creating infrastructure for program monitoring		
Organizational and individual assessment skills	Gathering information about and assessing the organizational context, including demographics, current practices, leadership structure/support, and relevant policies and procedures that could influence implementation and assessing stakeholders, interpersonal and group dynamics, and other factors	Communication
Developing a program monitoring system	Helping sites identify measures for assessing/monitoring provider productivity, program utilization, and outcomes; identifying, accessing, and obtaining data from existing databases; and developing/preparing feedback reports for monitoring, adapting, and improving the program	Administrative and project management, assessment, communication, engaging stakeholders, learning and fostering learning
Monitoring program implementation status	Continually observing implementation progress by reading and interpreting data in feedback reports; assessing program fidelity to evidence-based care, compliance with VA policy and fit with organizational context; and assessing implementation barriers and enablers	Assessment, developing a program monitoring system, problem identification/solving

**Fig. 1:**
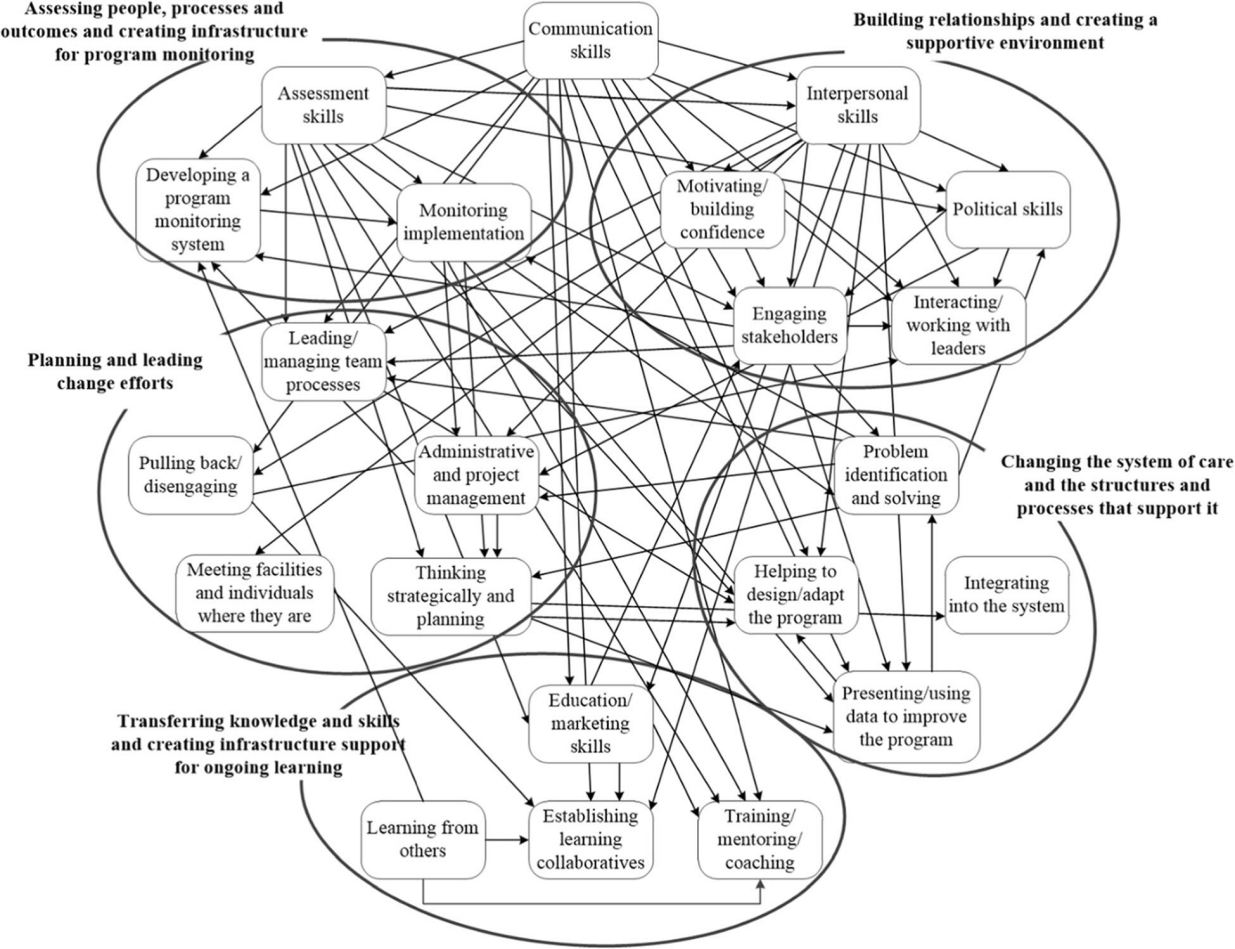
Relationships between implementation facilitation skills. Implementation facilitation skills are complex and overlapping. Arrows indicate that a skill is part of another skill or skillset. Facilitators may need the skill at the beginning of the arrow to operationalize the skill at the end of the arrow

With a few exceptions, implementation facilitation skills were not unique but overlapped with one another so that many of the skills included other skills. Figure 1 graphically represents these relationships, using arrows to indicate that a skill is part of another skill to which the arrow is pointing. We share this figure to illustrate the complexity of implementation facilitation skills and how they overlap but refer the reader to Table 1 for specific information about how each skill relates to other skills. Of note, communication, interpersonal, and assessment skills were part of many of the 22 skills we identified.

## Discussion

Facilitation, a multifaceted strategy that incorporates other interventions, is increasingly utilized to help healthcare systems, especially those that lack QI knowledge and resources, successfully implement evidence-based programs and policies. Scholars agree that facilitators need a wide variety of skills to apply and adapt these interventions to support successful implementation [13, 28–30]. However, few studies have attempted to identify skills facilitators need and no studies have documented implementation facilitation skills as they are being applied and transferred or explored the range of skills expert facilitators need for supporting implementation of complex innovations in challenging settings. In this study, we addressed that gap by identifying 22 complex implementation facilitation skills and providing descriptions of how these skills were operationalized. We also described the complex nature of facilitation skills and how skills clustered into 5 overarching conceptual groupings.

Although few studies have attempted to identify skills needed for implementation facilitation, a number of scholars [18, 37], including our own team [49], have compiled comprehensive lists of activities facilitators conduct to help sites implement innovations. It is likely the successful conduct of these activities will depend upon the skills that we identified in this analysis. For example, Dogherty and colleagues [37] identified and organized activities into a taxonomy of five categories (planning for change, leading and managing change, monitoring progress and ongoing implementation, and evaluating change). Harvey and Kitson [18] organized facilitation activities into four categories (clarifying and engaging, assessing and measuring, taking action and implementing, and reviewing and sharing). The facilitation activities these authors describe and categorize would likely require skills from two of our overarching skillsets: *planning and leading change efforts* and *assessing people, processes, and outcomes and creating infrastructure for program monitoring*.

There is further convergence between the skillsets we have identified and the facilitation activities that other scholars have described. For example, we identified a skillset concerning *building relationships and creating a supportive environment* in accordance with many implementation scholars who have acknowledged the necessity of building relationships with stakeholders to support implementation [13, 21, 22, 37]. Additionally, education and training strategies are widely utilized in implementation efforts and some scholars suggest that training and coaching staff is a core component of implementation [5]. For these efforts, facilitators need a skillset for *transferring knowledge and skills and creating infrastructure to support ongoing learning*, one of our skillset groups*.* Most lists of facilitation activities include problem identification and solving, designing and adapting an implementation plan, and utilizing data to improve implementation. For these activities, facilitators need a skillset for *changing the system of care and the structures and processes that support it*. Finally, literature clearly affirms the need for communication skills [37, 38, 50]. This study supports the work of other scholars but moves beyond their work to suggest that communication skills are part of many of the other skills identified in this study and thus a component of all of the larger skillsets.

In addition to conceptualizing what facilitators do as “activities,” we might also conceptualize what they do through the lens of implementation science more generally. Although facilitation is considered to be an implementation strategy [51], i.e., a method or technique “used to enhance the adoption, implementation, and sustainability of a clinical program or practice” [52], in reality, facilitators incorporate multiple other strategies into their efforts to support implementation [53, 54]. The Expert Recommendations for Implementing Change (ERIC) project created a taxonomy of discrete implementation strategies, one of which is facilitation [51]. Perry et al. [54] tested the ERIC taxonomy in multiple large studies that applied practice facilitation, “a more specific type of implementation facilitation,” to support implementation of evidence-based cardiovascular preventive care, and they further refined the ERIC taxonomy. The study found that facilitators did incorporate many other strategies into their efforts. Similar to literature exploring facilitation activities, skills we identified support facilitator’s application of other implementation strategies. For example, the ERIC strategy, “assess for readiness and identify barriers and facilitators,” will require that the facilitators have *organizational and individual assessment skills*; the strategy, “develop an implementation blueprint,” will require skills for *helping to design/adapt a program to meet local needs*.

We generally assume that skilled individuals have both explicit knowledge about what needs to be done (e.g., facilitation activities or implementation strategies to support implementation) and explicit and tacit knowledge of how to do it proficiently. Explicit knowledge can be codified, stored, and transferred easily through oral or written communications [55–57]. Tacit knowledge takes the form of beliefs, understandings, skills, and practices; has sometimes been called “know-how”; is generally acquired through experience and learning from others; and forms the basis for judgment and decision-making [56, 58, 59]. Providing novice facilitators with knowledge of facilitation activities and implementation strategies, their definitions, and principles or “rules” for applying them is necessary but insufficient for transferring the skills needed for facilitating implementation of innovations. Research into differences between novices and experts suggest that experts have a more highly organized knowledge base that allows them to intuitively recognize patterns between problems and solutions so that they can quickly respond with appropriate actions [60]. They utilize metacognitive strategies to select the appropriate activities likely to result in successful outcomes, monitor what they are doing, and quickly modify it as needed. Thus, the skills we identified in this study have both explicit and tacit dimensions. The journey from novice to expert facilitator will require the transfer of both. In our study, the expert facilitator transferred implementation facilitation skills directly to novices. Although iPARIHS framework developers suggest a model for transferring skills that involves a network of facilitators so that novices can be mentored and supported by peers and more experienced facilitators [61], they do not provide information about the methods that can be used to transfer complex implementation facilitation skills. Future work should address this gap.

Another key finding of this study is that implementation facilitation skills are very complex. Although other scholars have sought to identify and, to a limited extent, describe these skills, none have explored this complexity or its implications for training facilitators or for learning facilitation skills. By identifying and examining skills the external facilitator helped internal facilitators learn, both in depth and over time, and comparing task and behavioral components across skills, we were able to explore this complexity. Implementation facilitation skills include multiple activities and components. For example, an established body of communication research and theory suggests that communication skills include being able to proficiently perform many specific tasks and behaviors (e.g., listening, presenting information, clarifying and confirming, persuading, and asking and answering questions) [62]. None of these are simple tasks on their own. In addition to the complex nature of each of the identified skills, most facilitation skills are not unique or distinct. In this study, we found that many skills or components of skills are also components of other skills. For example, assessment skills, a set of skills widely held to be important for facilitating innovation implementation, include elements of communication skills (i.e., the ability to ask questions and clarify and confirm answers). Similarly, scholars describe interpersonal skills as having components of communication skills [63]. Scholars do not agree on all of the components of interpersonal skills [64], and the overlap between these two sets of skills is emphasized by the significant literature on interpersonal communication [65]. Thus, literature about these three skills supports their relationship to one another. Interestingly, one or more of these three skills, communication, interpersonal, and assessment, are components of almost every other set of skills identified in this study. Significantly, many scholars identify these three as core facilitation skills [29]. This study is the first to highlight this characteristic of implementation facilitation skills and contribute toward conceptual clarity about them. However, how to combine and apply such complex skills is largely tacit knowledge that has implications for transferring skills to novice facilitators, assessing skills in potential facilitators, and evaluating facilitators’ competency. In an upcoming project, we will be developing the processes for assessing skills and evaluating competency.

Finally, research and theory confirm that context influences implementation efforts [66, 67]. The iPARIHS framework supports the active role of facilitation in addressing the characteristics of context that can hinder or support implementation and also posits that there is interaction between facilitation and context [18]. Facilitators adapt what they do to fit local needs and conditions. This could involve spending more time on certain activities than on others. For example, where clinicians perceive that current practices are not problematic, there may be a need for a relatively higher level of initial marketing and education. Thus, at such a site, skills associated with these activities will be relatively more important. However, it may be possible that there are contextual influences that cannot be addressed by facilitation. In the larger project for this study, we concluded that facilitators would have difficulty being successful unless they had at least a moderate amount of leadership support and resources for implementation [16]. Despite skilled facilitation, one of the project’s community-based outpatient clinics was unable to implement PCMHI due to a lack of contextual supports. Because facilitation was provided for 2 years, facilitators had time to help the clinic’s parent VA medical center implement PCMHI; medical center leadership were then willing to work with facilitators to support implementation at the study site. Most facilitation interventions are of shorter duration and possibly less intensive than the one applied in the larger project. It may be that some contexts are so challenging that even skilled facilitators will be unable to engage leadership and obtain resources needed for successful implementation. Future research should explore contextual influences that need to be addressed for facilitation to be successful.

### Limitations and strengths

This study has a number of limitations that may affect transferability of study findings to other facilitation training efforts. First, there was only one expert transferring facilitation skills, and her training and background likely influenced what and how she trained the novice facilitators. Second, facilitators were supporting implementation of a policy initiative that included a mandate for implementation. This may have influenced the types of skills novice facilitators needed to learn. Because this study afforded us the opportunity to conduct an in-depth exploration, we sacrificed transferability of study findings to gain richness in the description of implementation facilitation skills. This richness will allow others to build on this work.

Additionally, there are many approaches to implementation facilitation. By design, the larger project utilized an intensive facilitation strategy in which facilitators did everything possible to maximize the successful implementation of a very complex program, Primary Care-Mental Health Integration, in clinics that would have experienced significant implementation challenges without facilitation. Thus, novice facilitators had to learn a broad range of complex skills with the goal of becoming expert facilitators over time. Some facilitation approaches are more restrictive (e.g., [21]); the innovation that facilitators are implementing may also be less complex than ours. There are likely core skills that all facilitators need but additional skills that may be required for complex or difficult implementation efforts. Identifying which are core facilitation skills and which are the ones needed for complex program implementation was beyond the scope of this study.

Findings should also be interpreted within the context of study strengths. This was the first study to document the skills transfer process as it occurred. Previous studies have identified implementation facilitation skills through literature reviews [20, 29] and eliciting facilitators’ retrospective reflections on the skills they utilized [13, 28, 37]. In addition to utilizing those methods, in this study, we also explored monthly documentation of an expert facilitator’s efforts to help novice facilitators learn facilitation skills. Using both deductive and inductive methods to analyze this data, we were able to create a longitudinal and rich description of expert implementation facilitation skills.

## Conclusions

This study identified and described a broad range of implementation facilitation skills that an expert transferred to initially novice internal regional facilitators to support implementation of a complex innovation. Because the innovation was multifaceted and implementing it required change across organizational structures and levels, as well as stakeholders, the list of skills we identified is comprehensive, though perhaps not complete. We also found that facilitation skills are complex and overlapping. Study findings have implications for planning and conducting implementation efforts utilizing facilitation as an implementation strategy, as well as for transferring and learning the skills to develop implementation facilitation expertise. To ensure fidelity to a planned strategy, planners should select facilitators who have the relevant skills or they should provide facilitators with opportunities to develop those skills [68]. Because implementation facilitation skills are complex, it is not likely experts can transfer them using only didactic methods. Future work should examine methods for transferring these skills. Additionally, future research should also explore core components of facilitation so early training could focus on ensuring that novice facilitators can learn skills needed to conduct those components.

We should not assume that because individuals have good communication and interpersonal skills, they will be good facilitators. This study suggests that even when new facilitators have those skills, they still may need significant help learning how to apply and adapt those skills to support implementation processes. Experts transferring facilitation skills for particular efforts or for building implementation capacity in healthcare systems need to understand the range of these skills, as well as their complexity, to help novice or less experienced facilitators become proficient in their application. Knowing that they may need a range of very complex skills may also help those developing facilitation skills to seek consultation and opportunities to learn. Finally, having a better understanding of skills facilitators need can also inform the efforts of healthcare system leaders wishing to build implementation facilitation capacity and policy designers who want to incorporate it as a policy tool.

## Supplementary information

**Additional file 1.** Skills code list development: source literature and materials. This file describes the source literature and publicly available training materials that informed the development of the qualitative code list applied in the study.

## Data Availability

The data generated by interviews with facilitators during this study are not publicly available due to the potential for compromising the privacy of study sites and their staff.

## Abbreviations

PCMHI: Primary care mental health integration
VA: Department of Veterans Affairs
