# Supplementary material for: From novice to expert: a qualitative study of implementation facilitation skills
Source: Implement Sci Commun. 2020 Feb 25;1:25. doi: 10.1186/s43058-020-00006-8 (PMC7427882; doi:10.1186/s43058-020-00006-8)
Supplement: Supplementary file 1 — Additional file 1. Skills code list development: source literature and materials. This file describes the source literature and publicly available training materials that informed the development of the qualitative code list applied in the study. [file 43058_2020_6_MOESM1_ESM.docx]

**Additional file 1**

**Skills Code List Development: Source Literature and Materials**

Note: The asterisk (*) indicates empirical studies that retrospectively identified facilitation skills

| **Skills** | **Literature** | **Practice Facilitation Training Materials** |
| --- | --- | --- |
| Administrative and project management | Being organized and prepared, particularly for meetings *[1]  Providing leadership: *[2, 3]  Non-specific: [4-7] | [8-12] |
| Assessment | Assessing context [4, 5, 13, 14]  Assessing implementation progress *[3]; [15]  Assessing needs of individuals and teams [4, 5]  Assessing organizational culture [16]  Diagnostic skills [4, 5]  Non-specific: *[3]; [17] | [9-11] [12] |
| Building capacity for and using data | Conduct data collection *[1, 3]  Expertise in acquiring and using data to drive improvement [18]  Fostering a culture of QI that includes use of performance data, creating capacity and expertise for process and outcome measurement, gathering data from performance reports, audits and/or outside sources and sharing data [15] | [8-10, 12, 19] |
| Change management | Knowledge and skills in the science of improvement, fundamental processes of practice involvement and organizational change, i.e., the basics of PDSA cycles for quality improvement (QI) [15]  Program planning and evaluation *[3]; [4]  Program implementation, development *[3]  Non-specific: *[3]; [5-7, 16, 17] | [9-11, 19] |
| Communication | Acquiring and processing information as well as being able to put arguments across [16]  Good listening skills [16, 20, 21]  Maintaining regular close contact, ensuring right people are informed, complex awareness (a kind of “communication sensitivity”) on multiple levels, persuasiveness [1]  Open to being contacted, friendly and outgoing [14]  Presentation skills [22]  Using open ended questions, asking reflexive questions, summarizing and checking understanding [21]  Non-specific: *[3]; [6, 18, 23, 24] | [8-10] |
| Customizing to local context | Non-specific: [15] |  |
| Education | Educate leaders about models of best practice; provide training to plan and implement innovations [15]  Non-specific: *[2] | [9, 10] |
| Helping others learn | Mentoring, teaching *[2, 3] and coaching *[2]  Role modeling to show how this is done *[3]  Supporting individual, team and organizational development and learning [4]  Non-specific: [5, 15] | [11, 12] |
| Interpersonal | Able to develop positive relationships [14]  Relational skills – making people feel comfortable to express themselves *[1]  Non-specific: *[2, 3]; [4, 6, 18, 24-27] | [8-12] |
| Marketing | How to locate sources of evidence, critically appraise literature, conduct data collection *[2, 3]  Non-specific: *[2]; [7] | [11] |
| Political skills | Political awareness of power relations and organizational decision-making processes [28]  Non-specific: [16] |  |
| Problem identification and solving | Characterizing and defining problems, defining barriers to good practice [29]  Non-specific: *[3]; [14, 16, 23] | [10, 19] |
| Providing support and encouragement | Providing encouragement and supporting gains [20]  Non-specific: *[2]; [30] |  |
| Stakeholder engagement | Ability to engender trust and engage/involve individuals in the change process *[2]  Overcoming resistance to change [4]  Understanding stakeholder perspectives *[3] | [8-10, 19] |
| Team development and management | Group dynamic and group leadership skills, making sure everyone is heard, assisting with shared decision-making and conflict resolution *[1]  Helping to set goals [15, 29]  Managing complex group dynamics, keeping team focused on task, maintaining momentum and team commitment [27]  Mediation *[2]  Negotiation *[1, 2]; [6, 29]  Non-specific: *[3]; [5, 17, 22, 24, 25] | [9-11] |
| Thinking and planning | Strategizing *[3]  Decision-making skills [16] |  |

References

1. *Dogherty EJ, Harrison MB, Baker C, Graham ID. Following a natural experiment of guideline adaptation and early implementation: a mixed-methods study of facilitation. Implement Sci*.* 2012;7:9. <https://doi.org/10.1186/1748-5908-7-9>.

2. *Dogherty EJ, Harrison MB, Graham ID, Vandyk AD, Keeping-Burke L. Turning knowledge into action at the point-of-care: the collective experience of nurses facilitating the implementation of evidence-based practice. Worldviews Evid Based Nurs*.* 2013;10(3):129-39. <https://doi.org/10.1111/wvn.12009>.

3. *Elnitsky CA, Powell-Cope G, Besterman-Dahan KL, Rugs D, Ullrich PM. Implementation of safe patient handling in the U.S. Veterans Health System: a qualitative study of internal facilitators' perceptions. Worldviews Evid Based Nurs*.* 2015;12(4):208-16. <https://doi.org/10.1111/wvn.12098>.

4. Harvey G, Fitzgerald L, Fielden S, McBride A, Waterman H, Bamford D, et al. The NIHR Collaboration for Leadership in Applied Health Research and Care (CLAHRC) for Greater Manchester: combining empirical, theoretical and experiential evidence to design and evaluate a large-scale implementation strategy. Implement Sci*.* 2011;6(1):1-12. <https://doi.org/10.1186/1748-5908-6-96>.

5. Seers K, Cox K, Crichton NJ, Edwards RT, Eldh AC, Estabrooks CA, et al. FIRE (facilitating implementation of research evidence): a study protocol. Implement Sci*.* 2012;7(1):25. <http://dx.doi.org/10.1186/1748-5908-7-25>.

6. Waterman H, Boaden R, Burey L, Howells B, Harvey G, Humphreys J, et al. Facilitating large-scale implementation of evidence based health care: insider accounts from a co-operative inquiry. BMC Health Serv Res*.* 2015;15(1):60. <https://doi.org/10.1186/s12913-015-0722-6>.

7. Rycroft-Malone J. The PARIHS Framework-a framework for guiding the implementation of evidence-based practice. J Nurs Care Qual*.* 2004;19(4):297-304. <https://doi.org/10.1097/00001786-200410000-00002>.

8. Knox L. Report on the AHRQ 2010 consensus meeting on practice facilitation for primary care improvement. Rockville, MD: Agency for Healthcare Research and Quality, 2010. Accessed 2 Feb 2014.

9. Coleman K, Pearson M, Wu S: A Practice Coaching Manual. In*.*: Rockville, MD: Agency for Healthcare Research and Quality; 2009. <http://www.improvingchroniccare.org/downloads/icic_practice_coaching_manual.pdf>. Accessed 6 Oct 2013.

10. Knox L, Taylor EF, Geonnotti K, Machta R, Kim J, Nysenbaum J, et al: Developing and running a primary care practice facilitation program: A how-to guide. In*.*, vol. Prepared by Mathematica Policy Research under Contract No. HHSA290200900019I TO5. AHRQ Publication No. 12-0011. Rockford, MD: Agency for Healthcare Research and Quality; 2011. <https://pcmh.ahrq.gov/sites/default/files/attachments/Developing_and_Running_a_Primary_Care_Practice_Facilitation_Program.pdf>. Accessed 24 Sept 2013.

11. Knox L, Brach C: Practice facilitation handbook: Training modules for new facilitators and their trainers. In*.* Agency for Healthcare Research and Quality, Rockville, MD; 2013. <http://www.ahrq.gov/professionals/prevention-chronic-care/improve/system/pfhandbook/index.html>. Accessed 17 Nov 2013.

12. Van Borkulo N, Coleman K: Practice facilitator's guide to visiting clinical teams. In*.*: Qualis Health and the MacColl Center for Health Care Innovation; Seattle, WA; 2011. <http://www.safetynetmedicalhome.org/sites/default/files/Facilitator-Guide-Site-Visit.pdf>. Accessed 15 Nov 2013.

13. Ellis I, Howard P, Larson A, Robertson J. From workshop to work practice: An exploration of context and facilitation in the development of evidence-based practice. Worldviews Evid Based Nurs*.* 2005;2(2):84-93. <https://doi.org/10.1111/j.1741-6787.2005.04088.x>.

14. Stetler CB, Legro MW, Rycroft-Malone J, Bowman C, Curran G, Guihan M, et al. Role of "external facilitation" in implementation of research findings: a qualitative evaluation of facilitation experiences in the Veterans Health Administration. Implement Sci*.* 2006;1:23. <https://doi.org/10.1186/1748-5908-1-23>.

15. Grumbach K, Bainbridge E, Bodenheimer T. Facilitating improvement in primary care: the promise of practice coaching. Issue Brief (Commonw Fund )*.* 2012;15:1-14.

16. Mccormack B, Garbett R. The characteristics, qualities and skills of practice developers. J Clin Nurs*.* 2003;12(3):317-25. <https://doi.org/10.1046/j.1365-2702.2003.00726.x>.

17. Nagykaldi Z, Mold JW, Aspy CB. Practice facilitators: a review of the literature. Fam Med*.* 2005;37(8):581-8.

18. Taylor EF, Machta RM, Meyers DS, Genevro J, Peikes DN. Enhancing the primary care team to provide redesigned care: The roles of practice facilitators and care managers. Ann Fam Med*.* 2013;11(1):80-3. <https://doi.org/10.1370/afm.1462>.

19. DeWalt DA, Powell J, Mainwaring B, McNeill J, Horowitz S, Margolis P, et al: Practice Coaching Program Manual. In*.*: Princeton, NJ: Aligning Forces for Quality (AF4Q), George Washington University Medical Center; 2010. <http://forces4quality.org/practice-coaching-program-manual>. Accessed 30 Oct 2013.

20. Bidassie B, Williams LS, Woodward-Hagg H, Matthias MS, Damush TM. Key components of external facilitation in an acute stroke quality improvement collaborative in the Veterans Health Administration. Implement Sci*.* 2015;10(1):69. <https://doi.org/10.1186/s13012-015-0252-y>.

21. Cross KD. An analysis of the concept facilitation. Nurse Educ Today*.* 1996;16(5):350-5.

22. Carroll L, Thirlwall M, Wilson A. Medical audit and the role of the facilitator. Int J Health Care Qual Assur*.* 1994;7(3):8-10.

23. Roberge P, Fournier L, Brouillet H, Hudon C, Houle J, Provencher MD, et al. Implementing a knowledge application program for anxiety and depression in community-based primary mental health care: a multiple case study research protocol. Implement Sci*.* 2013;8(1):26. <https://doi.org/10.1186/1748-5908-8-26>.

24. Thompson GN, Estabrooks C, Degner LF. Clarifying the concepts in knowledge transfer: a literature review. J Adv Nurs*.* 2006;53(6):691-701. <https://doi.org/10.1111/j.1365-2648.2006.03775.x>.

25. Harvey G, Loftus-Hills A, Rycroft-Malone J, Titchen A, Kitson A, McCormack B, et al. Getting evidence into practice: the role and function of facilitation. J Adv Nurs*.* 2002;37(6):577-88. <https://doi.org/10.1046/j.1365-2648.2002.02126.x>.

26. Burrows DE. Facilitation: A concept analysis. J Adv Nurs*.* 1997;25(2):396-404.

27. Cheater FM, Hearnshaw H, Baker R, Keane M. Can a facilitated programme promote effective multidisciplinary audit in secondary care teams? An exploratory trial. Int J Nurs Stud*.* 2005;42(7):779-91. <https://doi.org/10.1016/j.ijnurstu.2004.11.002>.

28. Kirk P, Broussine M. The politics of facilitation. Journal of Workplace Learning*.* 2000;12(1):13-22. <https://doi.org/10.1108/13665620010309756>.

29. Nzinga J, Ntoburi S, Wagai J, Mbindyo P, Mbaabu L, Migiro S, et al. Implementation experience during an eighteen month intervention to improve paediatric and newborn care in Kenyan district hospitals. Implement Sci*.* 2009;4(1):45. <https://doi.org/10.1186/1748-5908-4-45>.

30. Loftus-Hills A, Duff L. Implementation of nutrition standards for older adults. Nursing standard (Royal College of Nursing (Great Britain): 1987)*.* 1997;11(44):33-7.
